# Is *Clostridium perfringens* epsilon toxin associated with multiple sclerosis?

**DOI:** 10.1177/13524585231186899

**Published:** 2023-07-22

**Authors:** Richard W Titball, Nicholas Lewis, Richard Nicholas

**Affiliations:** Department of Biosciences, University of Exeter, Exeter, UK; Downing LLP, London, UK; Division of Brain Sciences, Department of Medicine, Imperial College London, London, UK

**Keywords:** *Clostridium perfringens*, epsilon toxin, multiple sclerosis

## Abstract

*Clostridium perfringens* epsilon toxin is associated with enterotoxaemia in livestock. More recently, it is proposed to play a role in multiple sclerosis (MS) in humans. Compared to matched controls, strains of *C. perfringens* which produce epsilon toxin are significantly more likely to be isolated from the gut of MS patients and at significantly higher levels; similarly, sera from MS patients are significantly more likely to contain antibodies to epsilon toxin. Epsilon toxin recognises the myelin and lymphocyte (MAL) protein receptor, damaging the blood–brain barrier and brain cells expressing MAL. In the experimental autoimmune encephalomyelitis model of MS, the toxin enables infiltration of immune cells into the central nervous system, inducing an MS-like disease. These studies provide evidence that epsilon toxin plays a role in MS, but do not yet fulfil Koch’s postulates in proving a causal role.

## Introduction

The bacterium *Clostridium perfringens* is found in the gastrointestinal tract of a range of animals and in the environment.^1^ Isolates of the bacterium are biotyped (A–G), according to the differential production of toxins.^2^ Type A strains often form part of the normal gut flora. Other biotypes cause disease in humans or in animals, because of the toxins they produce. Epsilon toxin is produced by *C. perfringens* types B and D strains and secreted from bacteria in the gut as protoxin^3^ which is activated by proteolytic cleavage.^4^

**Figure 1.**
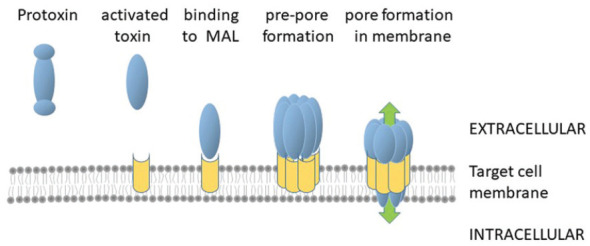
Pore formation by epsilon toxin. Protoxin is activated by the removal of N- and C-terminal peptides and the activated toxin binds to MAL receptor (shown in yellow) on susceptible cells. It is believed that seven monomers of the toxin assembled into a pre-pore which then undergoes a conformation change as it inserts into the membrane. The pore allows the unregulated movement of cations across the membrane (green arrows) leading to cell death.

After binding to the myelin and lymphocyte (MAL) protein receptor on target cell membranes, seven monomers of the toxin assemble into a cation-selective pore (Figure 1) which allows ion movement across the membrane, leading to cell death.^5,6^ Most cell types do not normally display MAL, and many cell lines are resistant to epsilon toxin.^7^ Resistant cells phenocopy toxin-sensitive cells when engineered to express the *MAL* gene.^5,8^ Naturally, susceptible cells include some epithelial and some endothelial cells (especially polarised epithelial cells), myelin-forming cells and some types of blood cells from some species, including humans.^9^

## Disease in animals caused by epsilon toxin

*C. perfringens* types B and D are the cause of a severe enterotoxaemia of animals, including domesticated livestock.^10^ The disease is often triggered by dietary changes, or after treatment with antibiotics which alter the gut flora, allowing the proliferation of *C. perfringens* to high levels and production of epsilon toxin in the gut.^11^ Enterotoxaemia is frequently fatal and is characterised by the short time between the first signs of disease and death.^12^ The signs of intoxication include body spasms, convulsions, incoordination, hazard roaming, head pressing and agonal struggling.^13^ Enterotoxaemia is of major economic significance to livestock rearing industries. Consequently, most livestock are immunised with a crude toxoid vaccine.

Toxin produced in the gut is translocated across the gut wall, entering the circulatory system.^14^ The subsequent disease pathology can differ in different animal species and depending on whether the disease occurs in the acute or sub-acute/chronic forms, but involvement of the brain is a consistent feature of disease. In experimentally infected sheep, which develop acute disease, a vasogenic oedema of the brain, heart and lungs has been reported.^15^ Accumulation of the toxin and kidney damage is frequently reported in sheep suffering from enterotoxaemia, but a recent study in experimentally infected sheep did not identify renal pathology.^16^ In rats dosed with epsilon toxin, brain lesions have been reported and additionally injury to the retinal microvascular.^17^

Most investigators have focussed on the effects of epsilon toxin in sheep, goats or mice.^12^ In sheep, and to a lesser extent goats, an early event is damage to the blood–brain barrier (BBB), associated with damage to vascular endothelial cells.^18^ In sheep, and if the dose of toxin is sufficiently high, a perivascular oedema has been reported which separates the end feet of astrocytes from blood vessels.^14,19^ Sheep or mouse astrocytes exposed to the toxin reportedly show upregulation of aquaporin 4 (AQP-4),^14,20^ and this might contribute to the oedema. Damage to the BBB may allow epsilon toxin to enter the brain, and a range of neuronal cells may then be affected (see cited review for detail).^14^ Oligodendrocytes cultured in vitro are susceptible to the toxin^21,22^ and epsilon toxin binds to oligodendrocytes in rat brain slices,^21^ but it is not clear if this cell type is affected in naturally intoxicated animals. Changes to the lamellation of myelin in the brains of intoxicated mice have been reported,^23^ and this could be prevented using a neutralising antibody against epsilon toxin.^22^ Lower doses of toxin can result in more profound histological changes with focal symmetrical encephalomalacia in the basal ganglia, internal capsule, thalamus, midbrain, medulla oblongata and cerebellar peduncles.^18^

## Sheep and multiple sclerosis in humans

The first suggestion of an association between exposure to epsilon toxin and the subsequent development of multiple sclerosis (MS) in humans was made by Murrell et al.^24^ His ideas were driven by a report that in a group of eight researchers working on swayback disease (a neurological disease of lambs), five subsequently developed MS.^25^ According to Dean et al.,^25^ the probability of this happening by chance was 1 in 10^9^. Since lambs are often colonised by *C. perfringens* strains producing epsilon toxin, he reasoned the researchers may have been exposed to the toxin. Murrell also suggested that hyperbaric oxygen used to treat MS might reduce the population of *C. perfringens* (a strict anaerobe) in the gut. In his study,^25^ Murrell et al.^24^ confirmed an association between the contact of humans with sheep or sheep products and the subsequent development of MS. However, he could not demonstrate experimentally that individuals who had developed MS had been exposed to epsilon toxin.

## MS and antibodies to epsilon toxin

Murrell et al.^24^ also attempted to measure antibodies to epsilon toxin in MS patients. He suggested that his inability to detect antibodies indicated the need for a more sensitive assay. It was another 40 years before two studies^26,27^ reported that antibodies that react with epsilon toxin are found in the sera of humans with clinically diagnosed MS (Table 1). These studies support the idea of past exposure to epsilon toxin in MS patients, but there are some unexplained findings. Wagley et al.^26^ found that many of the antibody responses were weak and it is unclear why so many sera from individuals, who did not have MS, reacted with epsilon toxin.

**Table 1.** Identification of antibodies that react with epsilon toxin, and *etx* gene present in faecal samples, from MS patients and matched controls.

Sample group	Number reactive/number tested					
	Serum antibody to epsilon toxin				*etx* gene in faecal samples	
	Rumah et al.^22 a^	Wagley et al.^21 a^	Wagley et al.^21 b^	Wagley et al.^21 c^	Maryam et al.^24 d^	Ma et al.^25 d^
MS patient	6/56 (11%)	14/61 (23%)	14/43 (33%)	26/61 (43%)	10/70 (14%)	19/31 (61%)
Matched control	0/60 (0%)	5/63 (8%)	5/32 (16%)	10/63 (16%)	0/70 (0%)	4/31 (13%)

MS: multiple sclerosis.

a Reactivity assessed by the western blotting against epsilon toxin.

b Reactivity towards peptides derived from epsilon toxin.

c Reactivity by the western blotting or towards peptides derived from epsilon toxin.

d Tested using polymerase chain reaction (PCR).

Another perplexing finding is that immune-reactive sera could not neutralise epsilon toxin, even if strongly reactive. All mammals tested to date develop toxin-neutralising antibodies after vaccination with an epsilon toxoid. Also, neutralising antibodies have been reported in a single human case of enteric disease associated with epsilon toxin.^28^ In the two recent serological surveys,^26,27^ sera from control subjects that recognised epsilon toxin also failed to neutralise the toxin. This indicates that the inability to produce neutralising antibodies is not specific to MS patients.^26^ These findings suggest that non-specific reactivity or cross-reactivity with another antigen cannot be excluded as an explanation of the reported antibody reactivity with epsilon toxin. It may also be significant that epitope mapping using an array of overlapping peptides spanning the protein indicates that different epitopes are recognised by human sera compared with immune sera from animals.^26^ Sera from rabbits immunised with a non-toxic variant of epsilon toxin consistently recognised peptides located predominantly in domains 1 and 3 of epsilon toxin.^26^ Sera from 33% of MS patients reacted with peptides, and these would be located in domains 1, 2 and 3 with few of the epitopes identified overlapping with those identified using rabbit immune sera.^26^ Some of the epitopes from MS patients were also identified in sera from control individuals who had not been diagnosed with MS. However, one epitope located in the membrane insertion loop was only recognised by sera from MS patients.^26^ This significance of these findings is not clear.

## MS and *C. perfringens* type B or D in the gut

While *C. perfringens* type A is common member of the gut flora, there are only two past reports of the isolation of *C. perfringens* type B or D from the gut of humans suffering from enteric disease.^11^ Neither individual was reported to show neurological signs of intoxication.^11^ More recently, a search of human gut metagenome data sets from 70 healthy volunteers identified *C. perfringens* generic genes, but not the epsilon toxin (*etx*) gene. These findings are consistent with *C. perfringens* as part of the normal gut flora,^26^ but suggest that strains producing epsilon toxin are rarely found in the normal human gut.

Rumah et al.^27^ first reported the isolation of a *C. perfringens* type B strain in the faeces of an MS patient, 3 months after the onset of symptoms. Later, a study from Iran reported that the *etx* gene was found in stools from 10 of 70 MS patients, but not in stools from 70 matched controls^29^ (Table 1). In a more recent study, where stool samples were processed to enrich for microbial DNA, *etx* was identified in 61% of MS patients (*n* = 31) compared to 13% (*n* = 31) of matched healthy controls.^30^ Also, when present, the *etx* gene was detected at significantly higher levels in MS patients than in controls.

## MS drugs inhibit growth of *C. perfringens*

Fingolimod, teriflunomide and dimethyl fumarate are oral medications used to treat MS and a recent study has shown that these drugs affect *C. perfringens* growth in vitro.^31^ However, these drugs are active towards a wide range of bacterial species^32,33^ and therefore is seems likely that they would change the gut microbiome. This might explain the reported reduction in the abundance of *Clostridia* in MS patients.^34^

## Human MAL is a receptor for epsilon toxin

In humans, MAL is expressed in a range of tissues including myelin-forming cells in the brain, in intestinal cells, cells lining ducts in the kidney and pancreas, and in cells in the thyroid and testis. Some human T-cells express MAL.^9,35^ A recent study demonstrated lysis of human erythrocytes,^36^ but not erythrocytes from other mammals. This is consistent with the finding that human erythrocytes, but not erythrocytes from other mammals, express MAL.^37^ The significance of this may be two-fold. First, fragility of erythrocytes may occur in MS,^35,38^ and this may be a consequence of exposure to epsilon toxin. If epsilon toxin was produced in the human gut and then entered the circulatory system, it is possible that it would bind to red cells. However, the picture might also be complicated by the finding that different isoforms of MAL exist on human cells,^9^ and it is possible that these have different affinities for epsilon toxin, meaning that transfer from low- to high-affinity receptors might occur in vivo.

## Sub-lethal doses of toxin in animals result in long-term damage

In animals suffering from enterotoxaemia, pathology occurs mainly in the brain, gut and heart. Although there is evidence of significant kidney pathology in cases of enterotoxaemia in sheep, this is believed to be largely a post-mortem phenomenon in toxin-damaged tissue.^13,16^ These findings are all consistent with MAL being expressed in these tissues. In contrast, in humans, cells from a wide range of tissues express MAL,^9,35^ but most of these tissues are not affected in MS.^39^

Sheep or mice exposed to lethal doses of toxin die rapidly, often within hours, showing signs of seizures.^40^ In these animals, a vasogenic oedema occurs. More interesting in the context of MS is the effect of sub-lethal doses of epsilon toxin on the brain, where mice show changes in motor functions, balance and social interactions.^41^ These signs can persist for at least a year, prompting comparisons with MS in humans.^41^ In mice and in sheep, a characteristic feature of exposure to sub-lethal doses of the toxin is necrosis in the basal ganglia, internal capsule, thalamus, midbrain, medulla oblongata and cerebellar peduncles (focal symmetrical encephalomalacia).^18^ In contrast, necrosis at these sites is not seen in MS.

## Epsilon toxin overcomes immune privilege in the experimental autoimmune encephalomyelitis model of MS

The experimental autoimmune encephalomyelitis (EAE) model in laboratory mammals is widely use to study MS and involves evoking an immune response to myelin-associated proteins.^42^ The subsequent permeabilisation of the BBB allows lymphocytes to cross the BBB causing damage to myelin-rich tissues in the central nervous system (CNS) with pathology that resembles that seen in MS in humans.^42,43^ Permeabilisation of the BBB is usually achieved using pertussis toxin, which binds to BBB endothelial cells, and the toxin may also have adjuvant properties by enhancing the immune response to myelin-associated antigens.^43^

A recent study by Ma et al.^30^ provides new insight by showing that epsilon toxin can substitute for pertussis toxin in the EAE model of MS. Moreover, the pathology seen in EAE mice which have been dosed with epsilon toxin more closely resembles MS than the pathology seen when EAE mice have been dosed with pertussis toxin.^30^ This suggests that the use of epsilon toxin in place of pertussis toxin would provide a more meaningful EAE model of MS. It may also indicate a role of epsilon toxin in the induction of MS in humans, by permeabilising the BBB and allowing autoreactive T-cells to evoke damage to myelin-bearing cells in the CNS.

## Conclusion

Over the past 50 years, a body of information has accumulated on the pathogenesis of enterotoxaemia in livestock animals, and we now have a good understanding of the key role that epsilon toxin plays in this disease. In diseased animals, *C. perfringens* types B or D become dominant members of the gut flora, with production of epsilon toxin in the gut. High doses of toxin cause the rapid death of animals, with minimal pathology in the brain other than a perivascular oedema.

In contrast, enteric disease caused by epsilon toxin in humans is very rare, even though human cells display the receptor for the toxin and isolated human cells expressing MAL are susceptible to the toxin. There may be a number of reasons for the low incidence of enterotoxaemia in humans. First, the overgrowth of *C. perfringens* producing epsilon toxin does not appear to occur in the human gut. This might be because in (herbivorous) animals, overgrowth is associated with a change from a starch poor to a starch rich plant-based diet, and the biology of the gastrointestinal tract is different in humans and in herbivores. Diet is believed to influence the risk of developing MS, and it may valuable to investigate how dietary changes influence the growth of *C. perfringens* in the gut. Second, cells expressing human MAL appear to be a less susceptible to the toxin than, for example, cells expressing sheep MAL,^44^ meaning that humans may be less susceptible to the toxin than other animals. Finally, unlike erythrocytes in livestock animals, human red cells express MAL and can bind toxin. Against this background, the reported effects of low doses of epsilon toxin in mice and sheep may be more relevant when considering the effect of the toxin in humans.

Several studies provide tantalising evidence that epsilon toxin plays a role in MS, but none of the studies reported to date fulfil Koch’s postulates in proving a causal role for the toxin. It is possible that epsilon toxin is a contributor to disease in some, but not all, individuals suffering from MS. The inability to detect antibodies to epsilon toxin in all MS patients and to isolate *C. perfringens* types B or D from the gut of MS patients might reflect the intermittent exposure of individuals to the toxin, and this makes it challenging to prove aetiology. It would be valuable to establish the temporal patterns of gut colonisation by *C. perfringens* strains producing epsilon toxin and determine whether the levels of antibody to epsilon toxin change over time. There is also a need to understand the nature of the antibody response to epsilon toxin in humans – does this antibody cross-react with epsilon toxin or is it toxin-specific antibody? Understanding why antibodies do not neutralise toxin activity is central to an understanding their specificity. Measuring the affinity of the antibodies towards epsilon toxin might provide insight into the likely specificity of the antibodies in human sera towards epsilon toxin.

The brain pathology associated with MS in humans and enterotoxaemia in animals are not similar, suggesting that epsilon toxin is not directly involved in tissue damage in MS in humans. However, epsilon toxin may play a role in overcoming the immune privilege associated with CNS tissues, by permeabilising the BBB.^30^ Like pertussis toxin, epsilon toxin may disrupt the BBB in EAE mice, allowing immune cells to enter the CNS. It also seems possible that toxins other than pertussis toxin and epsilon toxin, which target endothelial cells,^45^ could modify the permeability of the BBB. This raises the possibility that exposure to one of a number of toxins could serve to trigger the development of MS in individuals, although these toxins alone do not cause the disease. Further work is required to investigate this possibility.
